# Paroxysmal Autonomic Instability with Dystonia after Pneumococcal Meningoencephalitis

**DOI:** 10.1155/2012/965932

**Published:** 2012-10-11

**Authors:** Layal Safadieh, Rana Sharara-Chami, Omar Dabbagh

**Affiliations:** ^1^Division of Neurology, Department of Pediatrics and Adolescent Medicine, American University of Beirut Medical Center, P.O. Box 11-0236, Riad El Solh, Beirut 1107 2020, Lebanon; ^2^Division of Critical Care Medicine, Department of Pediatrics and Adolescent Medicine, American University of Beirut Medical Center, Beirut 1107 2020, Lebanon

## Abstract

*Streptococcus pneumoniae* is a common cause of bacterial meningitis, frequently resulting in severe neurological impairment. A seven-month-old child presenting with *Streptococcus pneumoniae* meningoencephalitis developed right basal ganglia and hypothalamic infarctions. Daily episodes of agitation, hypertension, tachycardia, diaphoresis, hyperthermia, and decerebrate posturing were observed. The diagnosis of *paroxysmal autonomic instability with dystonia* was established. The patient responded to clonidine, baclofen, and benzodiazepines. Although this entity has been reported in association with traumatic brain injury, and as a sequel to some nervous system infections, this is the first case, to our knowledge, associated with pneumococcal meningoencephalitis.

## 1. Introduction

*Paroxysmal autonomic instability with dystonia* (PAID) syndrome is a relatively uncommon complication of various central nervous system (CNS) injuries. It has been reported in association with severe traumatic brain injury, brain anoxia, subarachnoid and intracranial hemorrhages, midbrain glioma, and occasionally hydrocephalus [1, 2]. It is rarely encountered in patients with CNS infections. Two individual case reports of patients with tuberculous meningitis manifesting symptoms of PAID are described in the literature [3, 4]. Attacks of agitation, hypertension, fever, autonomic dysfunction, and extensor posturing are highly suggestive of PAID syndrome. Prompt recognition of this entity is crucial for the institution of proper and timely therapy.

## 2. Case Report

A 7-month-old previously healthy boy presented to the emergency department (ED) in status epilepticus of one hour duration characterized by continuous staring and generalized clonic movements. He was febrile (39 degrees Celsius), and his physical examination revealed a bulging anterior fontanel and nuchal rigidity with bilateral otitis media. Low grade fever was noted over the preceding week. The child was fully vaccinated except for the conjugated pneumococcal vaccine. Diazepam, phenytoin, and valproic acid were used in succession to achieve seizures control within 45 minutes from arrival to the ED. Ceftriaxone (100 mg/kg/day), vancomycin (60 mg/kg/day), and acyclovir (60 mg/kg/day) were empirically and promptly started. In view of recurrent apneas and seizures, the child required ventilatory support and remained intubated for the first fourteen days of hospitalization.

In the ED and prior to the pediatric intensive care unit admission, the workup consisted of the following: brain computed tomography scan (CT) that revealed bilateral osteomastoiditis. An electroencephalogram (EEG) could not be performed in the ED. His cerebrospinal fluid (CSF) analysis yielded 100 white blood cells/mm^3^ (82% polymorphonuclear leucocytes), 135 red blood cells/mm^3^, total protein of 3.2 g/L (reference value 0.1–0.5 g/L) and glucose of <3 mg/dL (reference value 35–80 mg/dL). Gram stain revealed abundant gram-positive diplococci. The blood sugar was 90 mg/dL; a complete blood cell count revealed 10200/mm^3^ white blood cells (reference value 4000–11000) and a c-reactive protein serum level of 487 mg/L (reference value <2.5 mg/L) with normal coagulation and liver function profiles. Forty-eight hours after admission, his blood and CSF cultures grew *Streptococcus pneumoniae* (*S. pneumoniae* serotype 19 a). Polymerase chain reactions performed on the CSF for herpes simplex virus and enterovirus were negative.

**Figure 1:**
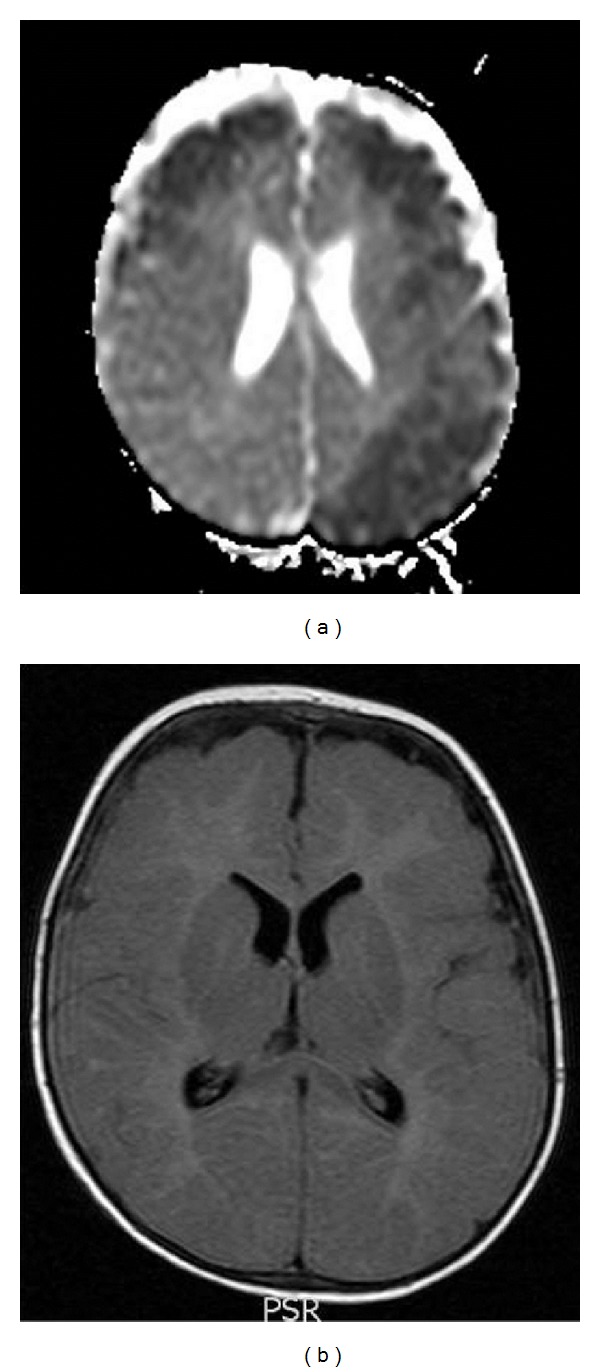
(a), (b) Brain magnetic resonance imaging one day after admission. (a) Axial apparent diffusion coefficient (ADC) map shows large areas of cortical and subcortical restricted diffusion in the frontal areas bilaterally, left parietal and left temporal regions, suggestive of encephalitis. (b) Fluid attenuated inversion recovery (FLAIR) sequence showing normal brain tissue.

One day later, the child developed near continuous choreiform movements of upper extremities and bicycling of lower limbs alternating with dystonia of all limbs that spontaneously revolved during sleep. A brain magnetic resonance imaging, angiography, and venography study (MRI, MRA, MRV) revealed large areas of cortical and subcortical restricted diffusion in the frontal areas bilaterally, left parietal and left temporal regions, suggestive of encephalitis, with no definite evidence for arterial or venous thrombosis (Figure 1(a)). No apparent lesions were demonstrated on routine MRI T1, T2, or fluid attenuated inversion recovery (FLAIR) sequences (Figure 1(b)). A 24-hour bedside video EEG study showed bilateral background slowing intermixed with sharp waves and sharply contoured theta activity. Several episodes of right-sided dystonic posturing were captured without any associated electroencephalographic correlates. Subsequently, phenytoin was discontinued and phenobarbital was added to valproic acid. Good therapeutic levels (phenobarbital level: 20–30 mg/L, valproic acid level: 50–70 mg/L) were maintained throughout his hospitalization.

The patient underwent bilateral myringotomy followed by left mastoidectomy. In view of MRI findings and precarious neurological condition, pulse methylprednisolone (30 mg/kg/day) was administered for three days but without significant improvement. Nasal gastric feeding and physical therapy were initiated.

Throughout the first week of hospitalization, the patient remained febrile with worsening of choreiform and dystonic movements. A chest radiograph showed right upper lobe consolidation and echocardiography was normal. Repeated cultures (blood, urine, and CSF) were negative. Based on the infectious disease team's recommendations, his antimicrobial regimen was continued for a total of 21 days and optimized to include cefepime, amikacin and caspofungin and vancomycin. His immune profile was normal. 

**Figure 2:**
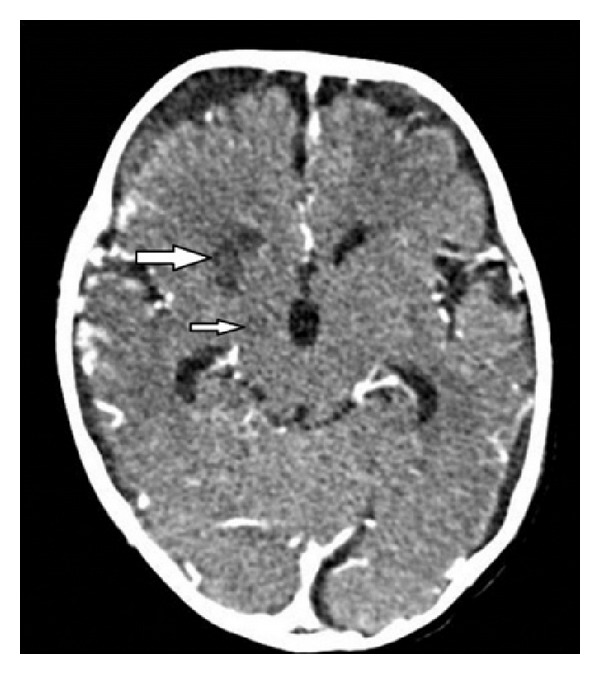
Enhanced brain computed tomography scan nine days after admission. Axial image through the basal ganglia shows hypodensities involving the right head of the caudate and anterior aspect of the putamen (thick white arrow) as well as the right hypothalamus (thin white arrow) representing subacute infarcts. In addition, there is development of bilateral hemispheric hygromas.

A follow-up brain CT scan performed at day nine of hospitalization showed an increase in size of the left parietal lobe and bilateral frontal and temporal lobes hypodensities, and appearance of new right basal ganglia (right head of the caudate and anterior aspect of the putamen), and right hypothalamus hypodensities (Figure 2). A follow-up lumbar puncture performed on the same day revealed a normal opening pressure, negative cultures, and improvement in the CSF parameters (48 white blood cells/mm^3^, protein of 1.29 g/L and glucose of 47 mg/dL), indicating a good response to ongoing therapies. 

Five days later, the patient developed daily episodes of severe agitation, hypertension (blood pressure 160/100 mmHg), sinus tachycardia (160 beats/minute), diaphoresis, and tachypnea with persistent hyperthermia (39 degrees Celsius) despite antibiotics and antipyretics and accompanied by decerebrate posturing. These episodes occurred at least twice a day lasting one to two hours and had no obvious electroencephalographic correlation. The diagnosis of *paroxysmal autonomic instability with dystonia* (PAID) was then established.

**Figure 3:**
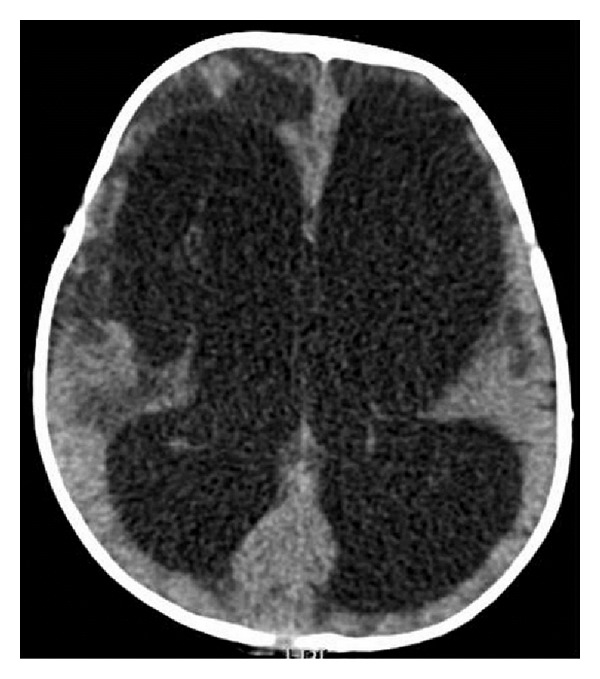
Nonenhanced brain computed tomography scan eight weeks after admission. Axial image shows significant nonobstructive hydrocephalus resulting in compression of cerebral parenchyma. There are hypodensities involving the periventricular regions and the frontal lobes bilaterally with associated cystic encephalomalacia, more on the right.

The episodes transiently responded to lorazepam and baclofen. Clonidine was started at 3 micrograms/kg/dose twice a day and increased to 5 micrograms/kg/dose, four times a day. The spells significantly responded to this therapeutic regimen, until eight weeks into the illness, when he developed episodes of severe irritability, sustained extensor posturing with generalized sweating, and bulging anterior fontanel. An urgent brain CT scan showed periventricular hypodensities, cystic encephalomalacia, and a nonobstructive hydrocephalus (Figure 3). 

The spells ceased one week after the insertion of a ventriculoperitoneal shunt. Two weeks later, clonidine was weaned off without further recurrence of the events. The child remained severely encephalopathic with minimal response to stimulation and continued to show severe upper motor neuron dysfunction in all four extremities.

## 3. Discussion

 PAID syndrome has been referred to under a variety of names, including *diencephalic seizures*, *paroxysmal sympathetic storm*, and *midbrain dysregulatory syndrome* [2]. The clinical manifestations of this syndrome are characterized by intermittent agitation, diaphoresis, hyperthermia, tachycardia, hypertension, tachypnea, hypertonia, and extensor posturing [5]. 

 Various mechanisms have been proposed for the paroxysmal dysautonomia seen in PAID syndrome. It is thought to be either secondary to dysfunction of the diencephalic autonomic centers (thalamus or hypothalamus) or disruption of their connections to other brain regions (cortical, subcortical, and brainstem), leading to loss of inhibitory inputs to sympathetic feedback loops and resulting in tachycardia, hypertension, hyperpyrexia, tachypnea, or diaphoresis [2, 6]. Hyperthermia may also result from either persistent muscle contraction or from hypothalamic dysfunction [5]. There is evidence that PAID syndrome also leads to loss of GABAergic inhibition of cortical projections, resulting in dystonic posturing [6]. The episodic nature of dysautonomia in PAID syndrome is probably related to triggering events such as fluctuations in intracranial pressure or stimulation of muscle mechanoreceptors, manipulation of endotracheal tube, oropharyngeal suctioning, and pain [1, 7]. All these PAID triggers were present in our patient, in addition to the subsequent development of nonobstructive hydrocephalus. The lumbar puncture performed around the time of onset of the dysautonomic spells revealed a normal opening pressure, and the brain CT scan showed right hypothalamic and basal ganglia infarcts without evidence of hydrocephalus.

 Since symptoms of PAID syndrome overlap with other serious conditions commonly found in the pediatric intensive care unit setting, a careful evaluation for alternative causes must be undertaken. Intermittent hypertension and tachypnea could be suggestive of intracranial mass, increased intracranial pressure, pain, or seizures. Fever, tachypnea, and diaphoresis often suggest an infection or a drug reaction. Dystonia or agitation may suggest increased intracranial pressure, inadequate analgesia, seizure activity or narcotic withdrawal [8]. There was no evidence of seizure activity, during the EEG recording, and therapeutic levels of anticonvulsants failed to alter the spells in our patient. In addition, he was not maintained on any anesthetic or muscle relaxant agents that could induce hyperthermia [2]. The latter persisted in spite of negative sepsis investigations and even after discontinuation of antibiotics ruling out drug-induced fever.

Various drugs have been used, either alone or in combination, to control the clinical features of PAID syndrome. No clear evidence suggests that one medication regimen is superior to another. After treating the underlying cause, adrenergic disinhibition has been successfully controlled by morphine, bromocriptine, a nonselective beta-blocker, clonidine (*α*2-adrenergic agonist) [1, 5, 6], or dexmedetomidine [8]. Additionally, benzodiazepines, intrathecal baclofen, and dantrolene have been used. Clonidine reduces blood pressure, helps control the sympathetic storm, and causes sedation [2]. The benzodiazepines, such as lorazepam, have sedating effects and muscle relaxant properties [2]. In our patient, the frequency and severity of the PAID syndrome events markedly decreased on clonidine, lorazepam, and oral baclofen.

*S. pneumoniae* meningoencephalitis may present as an occlusive, necrotising vasculitis, arterial thrombosis, and septic cortical thrombophlebitis [9]. The infarctions are usually confined to the gray matter. Basal ganglia are vulnerable for global and local ischemia and hypoxemia. However, Magnus et al. (2011) reported on an infant with *S. pneumoniae* meningoencephalitis who had acute vasculitis, leading to unusual basal ganglia necrosis [10]. Jorens et al. described in three adult patients (2005) and in one infant (2008) with pneumococcal meningoencephalitis widespread hyperintense lesions suggesting extensive central nervous parenchymal injury (predominantly the deep white matter) in the early course of the disease, presumably reflecting areas of ischemia with cytotoxic edema secondary to an immunologically mediated necrotizing vasculitis and thrombosis [9, 11]. Our patient had an early bilateral widespread grey white matter involvement with subsequent involvement of the right hypothalamus and basal ganglia.

PAID syndrome remains poorly understood and under-recognized phenomenon, despite its characteristic features. It is associated with significant morbidity, longer length of stay in rehabilitation services, and less favorable functional outcomes [3]. In addition, *S. pneumoniae* meningoencephalitis is associated with high mortality rate and severe neurologic complications including hearing loss, hydrocephalus, ischemic brain injury, and seizures [10]. Our patient is deaf and has severe neurologic impairment and significant developmental delay despite rehabilitation therapies. We believe that PAID syndrome is usually associated with severe neurological insult and thus the poor prognosis is most likely related to the primary insult.

The initial PAID manifestations observed in our patient were secondary to *S. pneumoniae* meningoencephalitis with involvement of the right hypothalamus and basal ganglia. The subsequent development of the hydrocephalus further exacerbated the PAID events. To our knowledge, this is the first case report of PAID syndrome in association with *S. pneumoniae* meningitis.

In conclusion, PAID syndrome remains a rare condition associated with CNS infections. Our report further expands the spectrum of disorders associated with this entity. Persistent dysautonomic dysfunction might result in further secondary brain injury as a result of significant persistent hyperthermia, increased energy expenditure, and increased intracranial pressure. A high index of suspicion leads to early recognition of the disorder, prompting institution of the appropriate therapies.

## References

[1] Srinivasan S, Lim CCT, Thirugnanam U (2007). Paroxysmal autonomic instability with dystonia. *Clinical Autonomic Research*.

[2] Blackman JA, Patrick PD, Buck ML, Rust RS (2004). Paroxysmal autonomic instability with dystonia after brain injury. *Archives of Neurology*.

[3] Ramdhani NA, Sikma MA, Witkamp TD, Slooter AJC, de Lange DW (2010). Paroxysmal autonomic instability with dystonia in a patient with tuberculous meningitis: a case report. *Journal of Medical Case Reports*.

[4] Antón JG, López Bayón J, López Fernández Y, Orive JP (2004). Autonomic dysfunction syndrome secondary to tuberculous meningitis. *Anales de Pediatria*.

[5] Wang VY, Manley G (2008). Recognition of paroxysmal autonomic instability with dystonia (PAID) in a patient with traumatic brain injury. *Journal of Trauma*.

[6] Goddeau RP, Silverman SB, Sims JR (2007). Dexmedetomidine for the treatment of paroxysmal autonomic instability with dystonia. *Neurocritical Care*.

[7] Boeve BF, Wijdicks EFM, Benarroch EE, Schmidt KD (1998). Paroxysmal sympathetic storms (diencephalic seizures) after severe diffuse axonal head injury. *Mayo Clinic Proceedings*.

[8] Goh KYC, Conway EJ, Darosso RC, Muszynski CA, Epstein FJ (1999). Sympathetic storms in a child with a midbrain glioma: a variant of diencephalic seizures. *Pediatric Neurology*.

[9] Jorens PG, Parizel PM, Demey HE (2005). Meningoencephalitis caused by *Streptococcus pneumoniae*: a diagnostic and therapeutic challenge. Diagnosis with diffusion-weighted MRI leading to treatment with corticosteroids. *Neuroradiology*.

[10] Magnus J, Parizel PM, Ceulemans B, Cras P, Luijks M, Jorens PG (2011). Streptococcus pneumoniae meningoencephalitis with bilateral basal ganglia necrosis: an unusual complication due to vasculitis. *Journal of Child Neurology*.

[11] Jorens PG, Parizel PM, Wojciechowski M (2008). *Streptococcus pneumoniae* meningoencephalitis with unusual and widespread white matter lesions. *European Journal of Paediatric Neurology*.

